# Artificial intelligence modeling of small molecule pharmaceutical solubility in supercritical fluid and comparative analysis under temperature and pressure variations

**DOI:** 10.3389/fchem.2026.1947884

**Published:** 2026-09-15

**Authors:** Haolin Wu, Hadil Faris Alotaibi, Ahmed Salah Fahem, Saeed Shirazian

**Affiliations:** 1 The Chinese University of Hong Kong, Hong Kong, China; 2 Department of Pharmaceutical Sciences, College of Pharmacy, Princess Nourah Bint AbdulRahman University, Riyadh, Saudi Arabia; 3 College of Engineering, University of Al Maarif, Al Anbar, Iraq; 4 Institute of Research and Development, Duy Tan University, Da Nang, Vietnam; 5 School of Engineering & Technology, Duy Tan University, Da Nang, Vietnam

**Keywords:** advanced manufacturing, machine learning, pharmaceutics, solubility, supercritical CO_2_

## Abstract

Enhancing the dissolution behavior of poorly water-soluble pharmaceuticals remains an important challenge in drug formulation and manufacturing, since limited aqueous solubility can substantially restrict therapeutic performance. Supercritical-fluid technologies provide a promising route for addressing this limitation, particularly through the production of nanoscale drug formulations with improved dissolution characteristics. In the present work, the ternary solubility of Nystatin in the SC-CO_2_–ethanol system was modeled as a function of temperature and pressure. Four regression approaches—K-nearest neighbors, linear regression, multilayer perceptron (MLP), and Theil–Sen regression—were examined as individual learners and subsequently incorporated into AdaBoost-based ensemble models. Model-specific hyperparameters were tuned using Glowworm Swarm Optimization whose population-based search strategy is suitable for exploring heterogeneous optimization spaces containing different parameter types. The developed ensemble configurations demonstrated strong agreement with the experimental solubility data, as reflected by high coefficients of determination and low root mean square errors. Among all evaluated models, the AdaBoost–MLP configuration provided the most favorable predictive performance, achieving an *R* ^2^ of 0.99412 together with the lowest root mean square error.

## 1 Introduction

There are various major challenges associated with pharmaceutical manufacturing among which poor solubility is a serious problem which needs to be addressed by proper manufacturing design and development (Chauhan, 2026; Pendam et al., 2025; Roosta et al., 2025; Zhang X.-R. et al., 2025). As such, the limited aqueous solubility of majority of small-molecule pharmaceutical compounds remains a major challenge in drug development, formulation, and manufacturing due to the complicated nature of the solubility. Poor solubility can constrain bioavailability, complicate dose selection, and reduce the efficiency of conventional separation and particle-engineering processes in pharmaceutical manufacture globally. Supercritical-fluid technologies offer a promising alternative because solvent properties can be tuned through relatively small changes in temperature and pressure. In particular, supercritical carbon dioxide (SC-CO_2_) is attractive for pharmaceutical applications because it is non-toxic, non-flammable, readily available, and easily removed after processing. Its relatively low critical temperature also makes it suitable for processing thermally sensitive compounds (Sodeifian et al., 2025a; Sodeifian et al., 2026; Wu et al., 2025; Zhang C. et al., 2025).

The process of supercritical fluid solvent can be employed to prepare pharmaceutical powder at submicron size thereby the solubility would enhance drastically. Despite these advantages, the solvent power of pure SC-CO_2_ is often insufficient for polar or high-molecular-weight pharmaceutical compounds. A substantial body of research has explored computational strategies for predicting the solubility of pharmaceutical compounds in neat supercritical CO_2_ (Altalbawy et al., 2025; He et al., 2025; Sodeifian et al., 2025b; Sodeifian et al., 2026). Nevertheless, the relatively limited solvent strength of pure SC-CO_2_ for many drug molecules motivates the use of organic modifiers to enhance solute–solvent interactions. The incorporation of a suitable co-solvent therefore represents an important extension of conventional supercritical-fluid processing, particularly for pharmaceutical systems in which solubility enhancement is required for efficient drug processing and formulation.

The addition of a polar co-solvent, such as ethanol, can increase solute–solvent interactions and improve the solubility of such compounds. Nystatin is an important antifungal drug whose processing may benefit from supercritical-fluid-based methods; however, its ternary solubility in SC-CO_2_–ethanol depends on interacting thermodynamic variables. Temperature and pressure affect fluid density, intermolecular interactions, and co-solvent behavior, producing nonlinear relationships that may be difficult to describe accurately using simple empirical correlations. Although experimental measurements remain indispensable for characterizing supercritical-fluid solubility behavior, generating such data typically requires substantial time, specialized equipment, and considerable experimental effort. Consequently, robust predictive tools can provide valuable support for process development and optimization by reducing reliance on extensive trial-and-error experimentation (Alwi et al., 2023). Despite growing interest in data-driven solubility prediction, comparatively fewer studies have focused on ternary pharmaceutical systems involving supercritical CO_2_ modified with an organic co-solvent. The present study contributes to this area by developing hybrid ML-based models for estimating drug solubility under such multicomponent supercritical conditions.

Earlier work has provided both experimental and computational foundations for analyzing Nystatin solubility in modified supercritical CO_2_. Sajadian et al. (Sajadian et al., 2023) generated ternary solubility measurements for the Nystatin–SC-CO_2_–ethanol system across a range of operating temperatures and pressures, and a later investigation reused this experimental basis to construct RF, ET, NU-SVR, and EPSILON-SVR predictors optimized through a metaheuristic search strategy (Majrashi et al., 2024; Sajadian et al., 2023). Together, these studies confirmed that data-driven methods can effectively represent the solubility behavior of this ternary system. The present work extends this modelling direction by examining a different set of regression learners within an AdaBoost ensemble framework and by employing GSO for model-specific hyperparameter optimization.

Machine learning (ML) has become increasingly useful for modelling complex chemical and pharmaceutical systems because they can learn nonlinear associations directly from experimental data (Balcázar et al., 2010; Bishop, 2006). In solubility prediction, these approaches can complement thermodynamic and empirical models when the available data are limited or when the governing relationships are difficult to specify analytically. Regression models are particularly suitable for this purpose because they estimate a continuous response variable from measured process conditions. The relative effectiveness of a regression method is inherently problem-dependent, as predictive performance is strongly influenced by dataset size, sampling distribution, measurement variability, and the complexity of the underlying input–output relationship. Consequently, no individual regression technique can be regarded as consistently superior across all datasets and modelling scenarios.

Classical thermodynamic and semi-empirical approaches remain important tools for correlating pharmaceutical solubility in supercritical CO_2_. Recent studies have employed EOS such as PR, SRK, and PC-SAFT, together with density-based and regular-solution-type correlations, to describe drug–SC-CO_2_ phase behavior (He et al., 2025; Sodeifian et al., 2025a; Zhang C. et al., 2025). These approaches provide useful thermodynamic interpretation but generally require model-specific parameters and compound-dependent calibration. Data-driven regression therefore represents a complementary strategy for capturing nonlinear solubility behavior directly from experimental observations. Notably, the experimental Nystatin data used in the present work were previously examined using empirical and semi-empirical correlations (Sajadian et al., 2023), providing an established conventional modelling context for the current machine learning analysis.

Recently, ML-based methods have been used for analysis of complicated systems because of their robust learning nature (Balcázar et al., 2010; Bishop and Nasrabadi, 2006). In the present study, the regression algorithms were selected deliberately to represent complementary modelling strategies rather than to provide an exhaustive comparison of all available ML techniques. Linear regression (LR) was included as an interpretable benchmark for approximately linear input–output relationships (Draper and Smith, 1998), whereas K-nearest neighbors (KNN) was selected as a local, non-parametric method capable of representing nonlinear patterns without imposing a predefined functional form (19, 20). Theil–Sen regression (TSR) provides a robust alternative with reduced sensitivity to outlying observations (Theil, 1950), while multilayer perceptron (MLP) offers greater flexibility for representing complex nonlinear dependencies. Taken together, these models enable a structured comparison of linearity, locality, robustness, and nonlinear learning capability in predicting Nystatin solubility.

Ensemble learning was incorporated to investigate whether the predictive capability of these complementary base learners could be further improved. Adaptive boosting (AdaBoost) iteratively places greater emphasis on observations that are difficult to predict and can consequently enhance the overall performance of the underlying regressors (Collins et al., 2002; Schapire, 2013; Schapire and Freund, 2012). Because the effectiveness of ensemble models depends on appropriate model configuration and hyperparameter selection, Glowworm Swarm Optimization (GSO), a population-based metaheuristic inspired by glowworm communication behavior, was employed for hyperparameter tuning (Kalaisel et al., 2017; Zhou et al., 2013). Its balance between local exploitation and global exploration makes it suitable for searching heterogeneous hyperparameter spaces containing continuous and discrete variables.

Accordingly, LR, KNN, MLP, and TSR were evaluated as base regression models for predicting the ternary solubility of Nystatin in the SC-CO_2_–ethanol system from temperature and pressure. Their corresponding AdaBoost configurations were subsequently examined, with GSO used for model-specific hyperparameter optimization. The resulting models were compared using *R* ^2^, RMSE, AARD%, and maximum error to determine their relative predictive performance within the investigated experimental range. Detailed mathematical and algorithmic descriptions of the individual methods are provided in the Methodology section.

## 2 Dataset and process description

The dataset used in this study was obtained from the experimental measurements reported by Sajadian et al. (Sajadian et al., 2023) for the ternary Nystatin–SC-CO_2_–ethanol system. A total of 28 experimental observations were considered, with temperature and pressure used as the two input variables and the measured ternary solubility of Nystatin as the prediction target. The experiments covered temperatures from 308 to 338 K and pressures from 12 to 30 MPa, while the ternary solubility ranged from 0.9 × 10^−5^ to 1.16 × 10^−4^. The dataset was randomly partitioned into 80% training and 20% testing subsets, corresponding to 22 and 6 observations, respectively. A fixed random seed (random_state = 42) was used to ensure reproducibility. Importantly, the same training and testing observations were used for all base regressors and their corresponding AdaBoost models, enabling a direct and unbiased comparison among the evaluated algorithms.

The same experimental source dataset was also employed in a previous ML investigation of the Nystatin–SC-CO_2_–ethanol system, in which pressure and temperature were used as predictors of ternary Nystatin solubility (Majrashi et al., 2024). The present work therefore reanalyzes this established experimental dataset and methodology using a different combination of base regressors, AdaBoost ensemble learning, and GSO-based hyperparameter optimization. Figures 1, 2 show the visualization of dataset for solubility for analysis of their variations (Majrashi et al., 2024).

**FIGURE 1:**
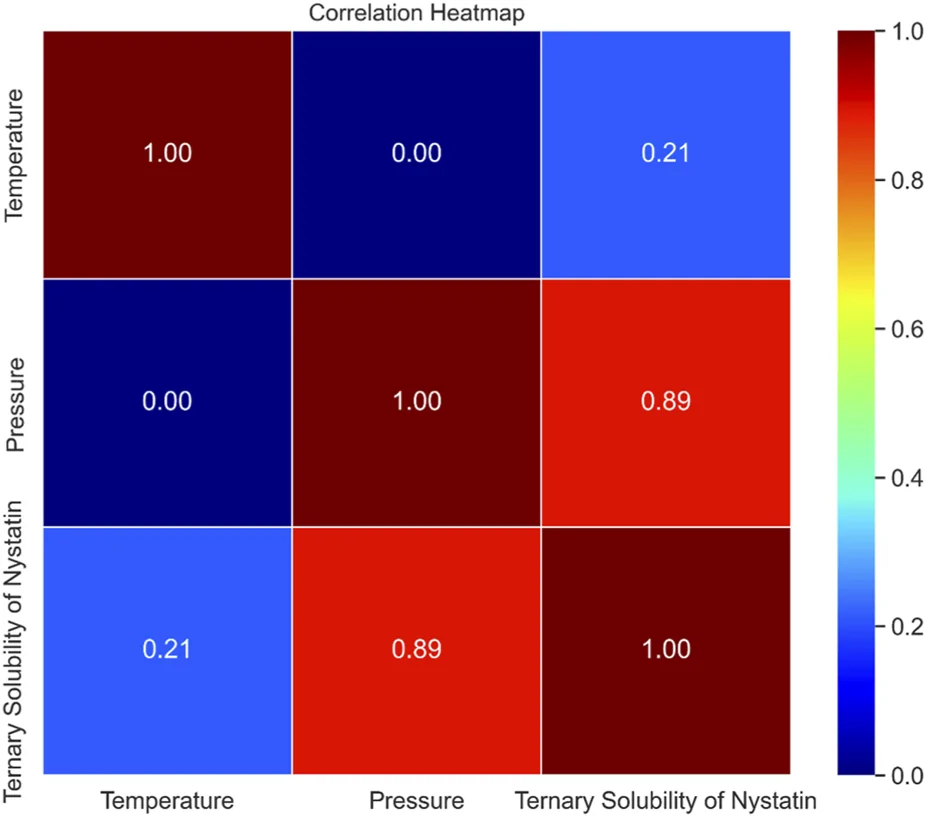
Pairwise correlation heatmap of variables (Majrashi et al., 2024).

**FIGURE 2:**
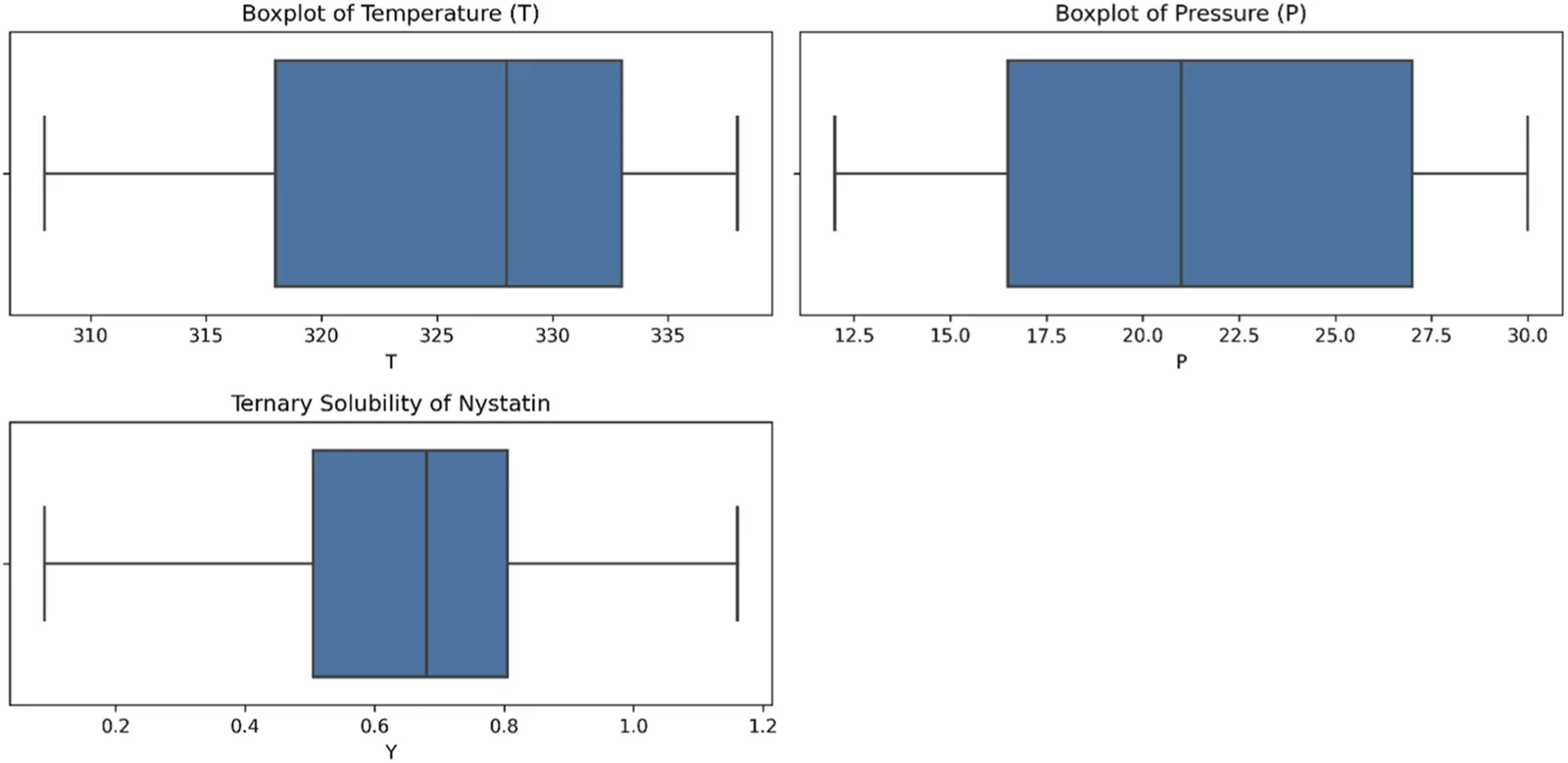
Box plots of variables.

To prevent information leakage, data partitioning was performed before any model preprocessing or hyperparameter optimization. The complete dataset was first divided into training (80%) and testing (20%) subsets using a fixed random seed (random_state = 42). Input normalization was then performed using parameters estimated exclusively from the training subset, and the same fitted transformation was subsequently applied to the test observations. The test-set information was therefore not involved in determining the preprocessing parameters. For each regression model, Glowworm Swarm Optimization was conducted independently using only the training data to identify the model-specific hyperparameter configuration. After optimization, the corresponding AdaBoost model was fitted using the training subset, whereas the held-out test subset was used only for final performance evaluation. The same train/test partition was maintained for all models to ensure a consistent and unbiased comparison.

The present study is a compound-specific modelling investigation rather than a multi-solute quantitative structure–property relationship framework. Nystatin is the only solute considered throughout the dataset; therefore, its molecular structure and associated descriptors remain invariant across all 28 experimental observations. Consequently, the variables responsible for the observed changes in solubility within this fixed ternary system are the investigated operating conditions, namely, temperature and pressure. Molecular descriptors or SMILES-based representations would become informative when multiple chemically distinct solutes are modelled simultaneously, which would require a substantially different dataset and modelling objective.

The experimental dataset analyzed in this work was obtained from the previously published Nystatin–SC-CO_2_–ethanol measurements reported in Ref. (Sajadian et al., 2023); therefore, no new experimental dataset was generated in the present study. The revised manuscript provides the complete data-partitioning procedure, preprocessing workflow, GSO configuration, and model-specific hyperparameter search spaces required to reconstruct the modelling protocol.

Temperature and pressure were selected because they are the principal operating variables systematically varied across the experimental observations used in this study and have direct physical relevance to supercritical-fluid solubility. Pressure strongly influences the density and solvent power of SC-CO_2_, whereas temperature affects both fluid density and the thermally dependent contribution of the solute to the overall solubility behavior. The present analysis was therefore intentionally restricted to variables that varied consistently across the available 28-point dataset. Additional molecular or process descriptors were not introduced because they were either invariant for this single-solute system or were not available as independently varying predictors across the complete dataset. Given the limited sample size, restricting the input space to the experimentally supported variables also avoids introducing unnecessary model complexity.

## 3 Methodology

### 3.1 Glowworm Swarm Optimization algorithm

Recent advances in ML have enabled the creation of sophisticated and accurate models for a variety of applications. Nevertheless, the success of these models is closely tied to the meticulous choice of hyper-parameters. These hyper-parameters are pivotal configurations that dictate the operation of a ML algorithm, influencing its effectiveness, rate of convergence, and capacity for generalization. The procedure for optimizing hyper-parameters involves identifying the most favorable combination of settings to achieve peak performance on a specific dataset (Kalaisel et al., 2017). GSO is a swarm-based metaheuristic inspired by the light-emitting behavior of glowworms, which use variations in bioluminescence to interact with nearby members of the population. In the optimization framework, candidate solutions are represented as individual glowworms that move within the search domain according to locally available fitness information. Through these neighborhood-based interactions, the algorithm can explore multiple promising regions of a complex search space and progressively move toward higher-quality solutions (Meng and Liu, 2023; Zhang, 2024a; Zhou et al., 2013).

Fundamentally, GSO functions by conceptualizing potential solutions as virtual glowworms that are dispersed throughout the hyper-parameter space. The brightness of each glowworm’s bioluminescence correlates with its fitness level, motivating them to explore in the direction of improved solutions (Meng and Liu, 2023). These virtual glowworms adjust their positions according to their individual fitness and the allure of their fellow glowworms, emulating the mutual attraction observed in actual glowworm colonies. This mechanism promotes both local and global exploration within the hyper-parameter space, empowering GSO to proficiently traverse intricate landscapes and uncover areas with promising potential. Figure 3 shows the workflow of GSO algorithm (Meng and Liu, 2023).

**FIGURE 3:**
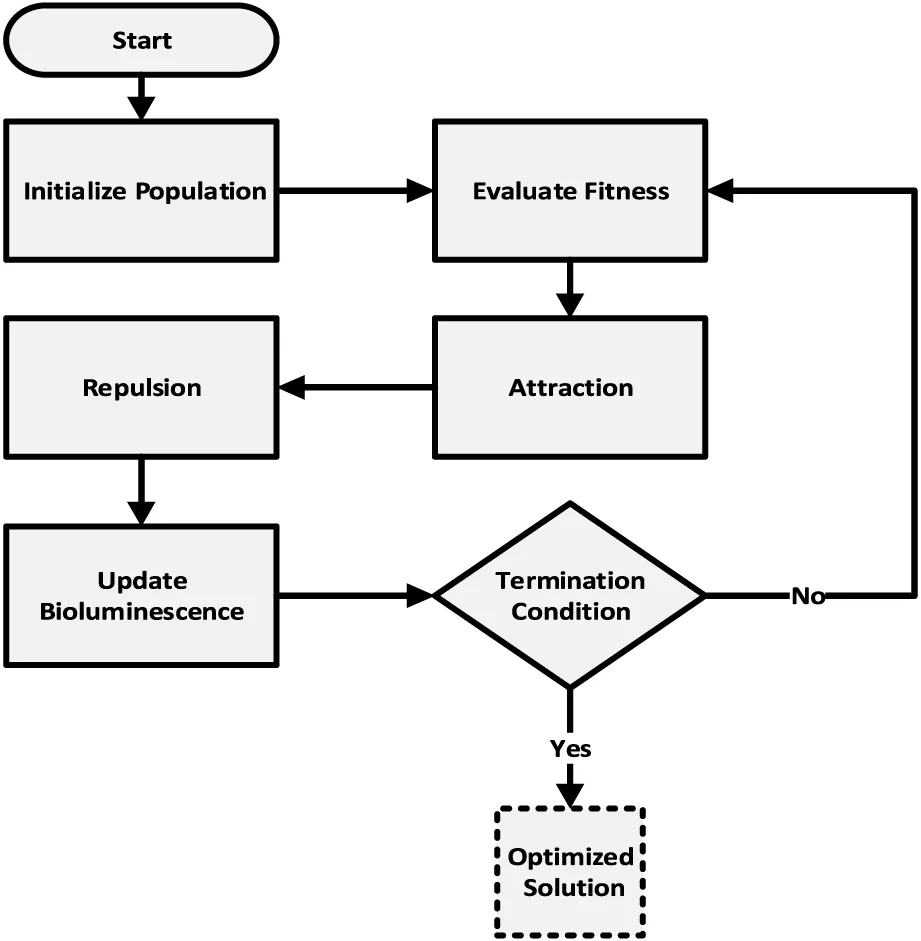
Glowworm swarm optimization algorithm.

For the present hyperparameter-optimization problem, GSO was selected because its luciferin-based search mechanism and adaptive local neighborhoods enable candidate solutions to explore multiple promising regions of the search space while maintaining a balance between local exploitation and broader exploration (Kalaisel et al., 2017; Zhou et al., 2013). This feature is particularly useful in the present study because the optimized models contain heterogeneous hyperparameters, including continuous, discrete, and categorical choices. Compared with other population-based metaheuristics, such as ant-colony or bee-colony approaches, GSO provides a distributed neighborhood-based search strategy that is well suited to locating multiple promising regions rather than forcing the population toward a single search trajectory. However, no direct benchmark against these alternative metaheuristics was performed in the present study; therefore, GSO is not claimed to be universally superior, but rather was selected as an appropriate and flexible optimizer for the current mixed hyperparameter-search problem.

For hyperparameter optimization, GSO was implemented with a population of 30 glowworms and a maximum of 50 iterations. The optimization was terminated after completion of the predefined 50 iterations, with no additional convergence tolerance imposed. The standard GSO control parameters were set to a luciferin decay constant of 0.4, a luciferin enhancement constant of 0.6, and a neighborhood-range adaptation coefficient of 0.08. The objective function was the RMSE calculated using the training data, and lower RMSE values represented higher-quality candidate solutions. Importantly, GSO was executed independently for each regression family so that the hyperparameter configuration of one model did not influence the optimization of another model. As described in the data-processing workflow, the held-out test observations were not used during GSO optimization.

### 3.2 Adaptive boosting regression (adaboost)

Adaboost is a powerful approach which excels in boosting the predictive capabilities of weak regression method in machine learning. Adaboost is renowned for its ability to transform a set of mediocre base models into a robust and accurate ensemble (Schapire, 2013; Schapire and Freund, 2013).

The central concept underlying Adaboost is the iterative training of base models, with a focus on those instances where the previous models have exhibited deficiencies. It assigns varying weights to data points, with higher emphasis on misclassified samples in each iteration, thus enabling the subsequent models to concentrate on rectifying the previously misclassified instances. This adaptability and focus on challenging cases make Adaboost particularly effective.

Mathematically, the core principle of Adaboost can be expressed as follows:

To include a dataset by *N* data-points $\left(x_{1},y_{1}\right),\left(x_{2},y_{2}\right),\ldots ,\left(x_{N},y_{N}\right)$ , where $x_{i}$ represents the input features and $y_{i}$ signifies the target variable. Adaboost aims to find an ensemble $F\left(x\right)$ of base models $f_{m}\left(x\right)$ , where *m* ranges from 1 to *M*, such that the weighted sum of their outputs minimizes the exponential loss (Collins et al., 2002). The mathematical formulations used for the predictive models are presented in Equations 1–3.

$$F\left(x\right)=\sum_{m=1}^{M}\alpha_{m}f_{m}\left(x\right)$$ (1)

Here, $\alpha_{m}$ represents the weight assigned to the *m*th base model, and $F\left(x\right)$ combines their predictions. Minimization is the main goal of the following exponential loss relation (Collins et al., 2002):

$$L\left(F\right)=\sum_{i=1}^{N}e^{{-y}_{i}F\left(x_{i}\right)}$$ (2)

Adaboost effectively adjusts the weights $\alpha_{m}$ and the importance of each sample to minimize this loss function. The final ensemble is derived by aggregating the individual base models, weighed by $\alpha_{m}$ , to produce a strong predictive model that has learned to focus on challenging instances and adapt to their requirements.

In essence, Adaboost excels in regression tasks by iteratively improving upon the weaknesses of its constituent models, adapting to intricate patterns in the data, and ultimately producing a highly accurate ensemble capable of tackling a wide array of real-world problems. Its ability to mitigate overfitting, adapt to noisy data, and generalize well makes it a preferred choice in the realm of regression modeling.

The hyperparameter search was model-specific. For the AdaBoost ensemble, the number of boosting estimators was explored from 20 to 150 and the learning rate from 0.01 to 1.0. For KNN, the number of neighbors was searched from 2 to 10, together with uniform or distance-based weighting and Minkowski distance orders of 1 or 2. For MLP, the number of hidden neurons was varied from 5 to 50, the regularization coefficient from 10^−6^ to 10^−2^, and the initial learning rate from 10^−4^ to 10^−1^, with tanh and ReLU considered as activation functions. For TSR, the number of subsamples was varied from 3 to 10 to control the robustness–efficiency trade-off. LR was retained in its standard least-squares form with an intercept because it has no comparable nonlinear model-specific tuning parameters; therefore, only the AdaBoost-level parameters were optimized for ADA-LR. All model-specific searches were conducted separately using the same training partition.

### 3.3 Linear regression

A widely employed and straightforward machine learning model for solving regression problems is LR. It aims to build a linear connection between input features and a continuous target variable. LR’s simplicity allows for easy interpretation and efficient training (Groß, 2003).

In LR, we seek to predict an output (Y) based on input features ( $X_{1},X_{2},\ldots ,X_{n}$ ) by determining the best-fit line. The fitted relationship is parameterized by a set of regression coefficients, $\beta_{0},\beta_{1},\beta_{2},\ldots ,\beta_{n}$ , which are estimated so that the discrepancy between the model predictions and the observed response values is minimized.

One of LR’s strengths is its interpretability. The coefficients (β) reveal the nature and strength of relationships between features and the target. Positive coefficients indicate positive correlations, while negative coefficients signify negative correlations. This interpretability is valuable for understanding feature impacts.

Despite its interpretability and computational simplicity, LR is inherently constrained by its assumption of a linear dependence between predictors and the response. Such an assumption may be inadequate for complex systems characterized by curvature or variable interactions. In these cases, more flexible nonlinear approaches, including MLP-based models and ensemble learning techniques, may provide a more suitable representation of the underlying data structure.

### 3.4 K-nearest neighbors

A versatile machine learning model employed for regression tasks is KNN. Unlike linear regression, KNN operates on the principle of similarity. It predicts the target value for a data point by considering the values of its k-nearest neighbors, where “k” is a user-defined parameter (Cover, 1968; Kramer and Kramer, 2013).

In KNN regression, predictions are generated from the response values of observations located closest to a query point in the feature space. Depending on the selected weighting strategy, neighboring targets may contribute equally or according to their relative distance from the query sample. Because the prediction is determined locally rather than through a predefined global equation, KNN can accommodate nonlinear patterns in the data.

An important advantage of KNN is its model-free structure. The method does not require an explicit assumption regarding the mathematical form of the relationship between the predictors and the target variable, which makes it applicable to a broad range of regression tasks. Its underlying procedure is also conceptually straightforward and comparatively easy to implement.

The performance of KNN, however, depends strongly on its configuration. In particular, the number of neighbors (k), governs the balance between local sensitivity and smoothing: very small values may make predictions more susceptible to noise, whereas excessively large values can suppress meaningful local variations. The selected distance metric can likewise influence which observations are regarded as similar and, consequently, affect the resulting predictions.

### 3.5 Multilayer perceptron

A feedforward neural-network model widely used for regression is MLP because of its ability to approximate complex relationships between explanatory variables and continuous responses. Its structure is composed of successive layers of connected computational units, through which information is transformed using learned weights and nonlinear activation functions. This layered representation enables the network to capture interactions and nonlinear dependencies that may be difficult to describe using simpler regression formulations.

Mathematically, MLP can be expressed as follows (Ramchoun et al., 2016):

$$\hat{Y}=f_{out}\left(f_{n}\left(X\cdot W_{n}\right)\cdot W_{out}\right)$$ (3)

Here, *X* represents the input data, $W_{h}$ and $W_{out}$ are the weight matrices of the hidden and output layers, respectively. The functions $f_{h}$ and $f_{out}$ denote activation functions applied to the hidden and output layers, which introduce non-linearity into the model. Common nonlinear activation functions used in neural-network architectures include the logistic sigmoid, hyperbolic tangent (tanh), and rectified linear unit (ReLU), each of which transforms neuronal inputs differently and influences the network’s learning behavior.

The architecture of MLP allows it to automatically learn and adapt to the underlying data patterns during training. The backpropagation algorithm, combined with gradient descent optimization, adjusts the weights to minimize a chosen loss function, typically Mean Squared Error (MSE) for regression problems.

The universal approximation theorem is a prominent advantage of the MLP model. It asserts that a feedforward neural network consisting of a single hidden layer has the capability to approximate any continuous function with arbitrary precision. This property underscores the MLP’s capability to model a wide range of data distributions.

Moreover, MLPs can be customized in terms of the quantity of hidden layers and neurons per layer, enabling flexibility to adapt to the complexity of the regression task at hand. However, this flexibility also introduces challenges in hyperparameter tuning, which can be effectively addressed by techniques like cross-validation and, as demonstrated in our study, GSO.

### 3.6 Theil-sen regression

Rather than relying exclusively on conventional least-squares fitting, TSR method derives its regression coefficients from the median of slopes calculated across pairs of observations. This median-based estimation procedure improves resistance to outliers and makes TSR particularly useful when the dataset may contain influential or noisy measurements (Theil, 1950).

TSR estimates the regression tendency by taking the median of slopes obtained from pairs of observations, rather than depending on a least-squares criterion. This median-based formulation reduces the influence of extreme or atypical data points and generally yields a more resistant estimate of the underlying relationship.

A major benefit of this approach is its reduced sensitivity to noise and outliers. Because isolated observations have less leverage over the fitted relationship, TSR can maintain stable predictive behavior even when some measurements depart noticeably from the dominant pattern in the dataset.

Another benefit of TSR is its simplicity and ease of interpretation. The model doesn’t involve complex mathematical equations or assumptions about the data distribution.

However, TSR also has limitations. It may not perform as well as some other regression methods when the data adheres strictly to a linear relationship. In such cases, parametric models like Linear Regression may provide more accurate predictions.

## 4 Results and discussion

This section assesses the predictive performance of the developed models for ternary Nystatin solubility in the SC-CO_2_–ethanol system. Model accuracy was quantified using the coefficient of determination (*R* ^2^), root mean square error (RMSE), average absolute relative deviation (AARD%), and maximum prediction error. Together, these indicators provide complementary measures of goodness of fit and prediction deviation, enabling comparison among the optimized model configurations and evaluation of the role of GSO-based hyperparameter tuning. The predictive performance of each optimized boosted model is summarized in Table 1.

**TABLE 1:** Performances of the boosted optimized models.

Model	R-squared	RMSE	AARD%	Maximum error
ADA-KNN	0.97038	3.0704E-02	6.25	5.50000E-02
ADA-LR	0.96917	3.4264E-02	5.03	4.73443E-02
ADA-MLP	0.99412	1.5282E-02	1.82	3.20309E-02
ADA-TSR	0.9646	3.6806E-02	6.79	5.02516E-02

The predictive performance of the models was evaluated using the coefficient of determination (*R* ^2^), root mean square error (RMSE), average absolute relative deviation percentage (AARD%), and maximum error. *R* ^2^ measures the proportion of variation in the experimental response that is reproduced by the model, with values closer to 1 indicating better agreement between predicted and observed values. RMSE quantifies the overall magnitude of prediction errors while giving greater weight to larger deviations; therefore, lower RMSE values indicate better predictive accuracy. AARD% expresses the average absolute prediction error relative to the experimental values as a percentage, providing a scale-normalized measure of model deviation, for which lower values are preferable. Maximum error represents the largest individual prediction deviation and provides an indication of the worst-case prediction error.

Among the investigated configurations, ADA-MLP delivered the most favorable point-estimate performance, attaining an *R* ^2^ of 0.99412 together with the lowest RMSE among the evaluated models. Figure 4 compares the experimental and model-predicted solubility values for this configuration. The concentration of data points close to the identity line indicates close agreement between measured and predicted responses over the investigated range, although this visual agreement alone should not be interpreted as definitive evidence of the absence of systematic prediction bias.

**FIGURE 4:**
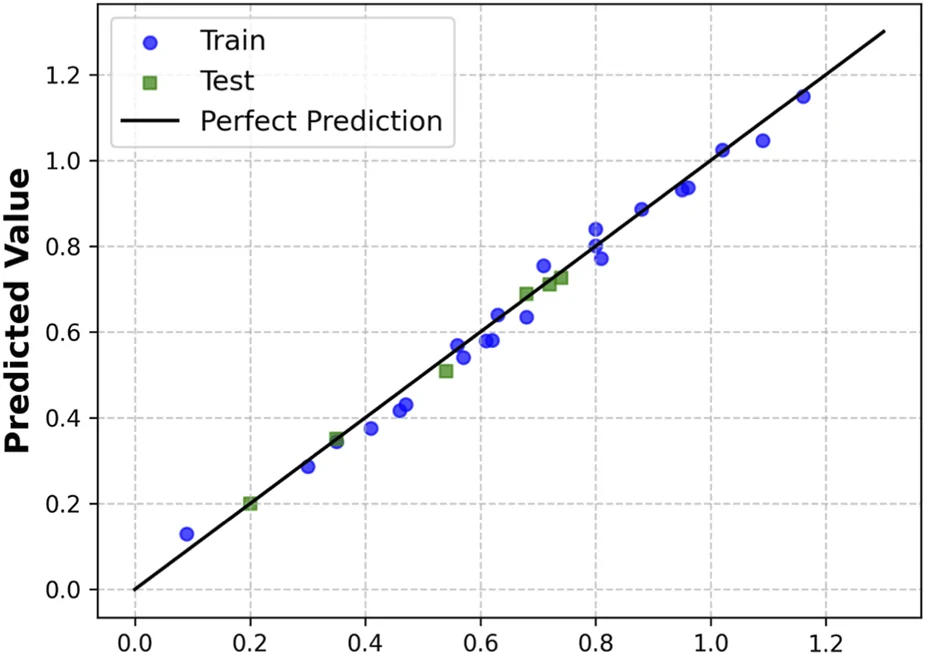
ADA-MLP model: Actual Vs. Predicted values.

The high *R* ^2^ obtained for ADA-MLP was interpreted with caution because the dataset is relatively small. Several procedural safeguards were adopted to reduce the risk of overfitting. The test subset was isolated before preprocessing and hyperparameter optimization, normalization parameters were estimated only from the training observations, and GSO tuning was conducted exclusively using the training subset. The final model was then evaluated on the previously unseen test observations. Accordingly, the reported performance should be interpreted as predictive accuracy within the investigated Nystatin–SC-CO_2_–ethanol domain rather than evidence of unrestricted generalization. Nevertheless, given the limited number of experimental observations, further validation using larger independent datasets or repeated resampling would be valuable for a more comprehensive assessment of generalization.

Among the evaluated models, ADA-MLP achieved the most favorable point-estimate performance, with the highest *R* ^2^ and the lowest RMSE, AARD%, and maximum error under the adopted train/test partition. Because the present analysis was based on a single fixed data partition and did not include repeated resampling or paired statistical testing, these differences should be interpreted as comparative predictive performance rather than evidence of statistically significant superiority. A more extensive statistical comparison based on repeated cross-validation, bootstrap confidence intervals, or paired error-based significance testing would provide a stronger assessment of between-model performance differences and is recommended for future work.

The differences among the four models can be interpreted in terms of their underlying learning characteristics. The comparatively favorable point-estimate performance of ADA-MLP is consistent with the ability of MLP to represent smooth nonlinear interactions between temperature, pressure, and solubility. This flexibility is particularly relevant to the present system because the effect of temperature is not strictly linear and may interact with pressure through changes in fluid density and solute-related thermal effects. AdaBoost can further emphasize observations that are more difficult for the base learner to reproduce, while GSO provides model-specific hyperparameter tuning; together, these features may help the MLP-based ensemble accommodate the observed nonlinear response more effectively. Nevertheless, given the limited dataset size, this interpretation is restricted to the investigated experimental domain.

ADA-KNN also produced strong predictive performance because its local, similarity-based structure can interpolate effectively among neighboring temperature–pressure conditions. However, predictions are determined by a finite set of nearby observations, which can generate locally stepped or less smooth responses and reduce flexibility in more sparsely represented regions. ADA-LR performed reasonably well because a substantial portion of the pressure–solubility relationship follows an approximately monotonic and near-linear trend within the investigated range; however, its linear structure limits its ability to represent curvature and interaction effects. ADA-TSR provides robustness by relying on median-based slope estimation and is therefore less influenced by unusual observations, but this robustness is obtained at the expense of some flexibility in reproducing subtle nonlinear variation. The observed ranking of the models is therefore consistent with a trade-off among nonlinear flexibility, local interpolation, linear simplicity, and robustness rather than reflecting accuracy differences alone.

The ADA-KNN and ADA-LR configurations also exhibited favorable predictive behavior, as reflected by their relatively high *R* ^2^ values and comparatively low RMSE levels. These results indicate that both models were able to reproduce the measured solubility response with reasonable accuracy within the investigated domain. The correspondence between experimental and predicted values for ADA-KNN and ADA-LR is illustrated in Figures 5, 6, respectively.

**FIGURE 5:**
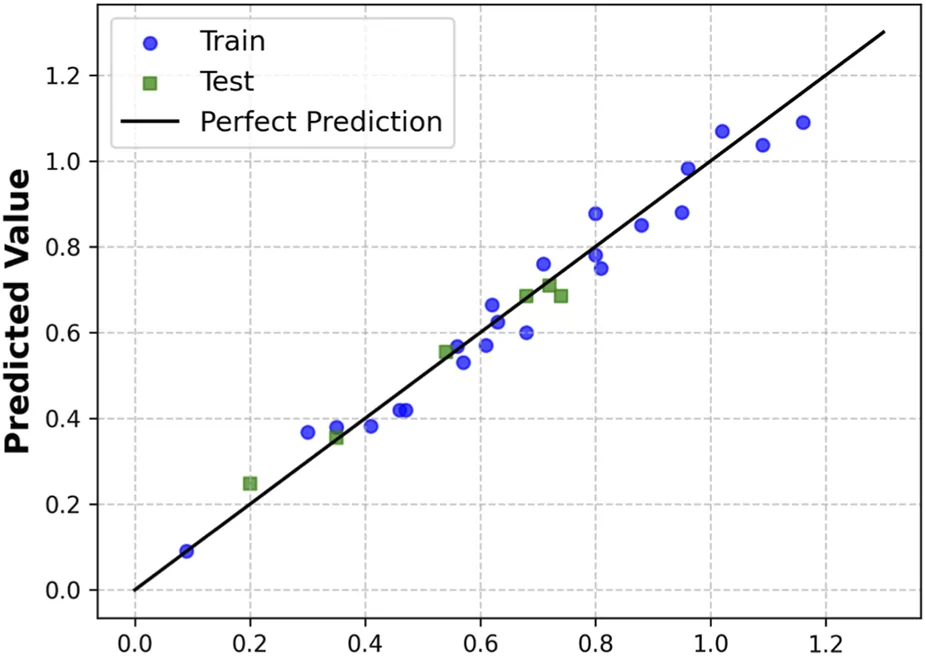
ADA-KNN model: Actual Vs. Predicted values.

**FIGURE 6:**
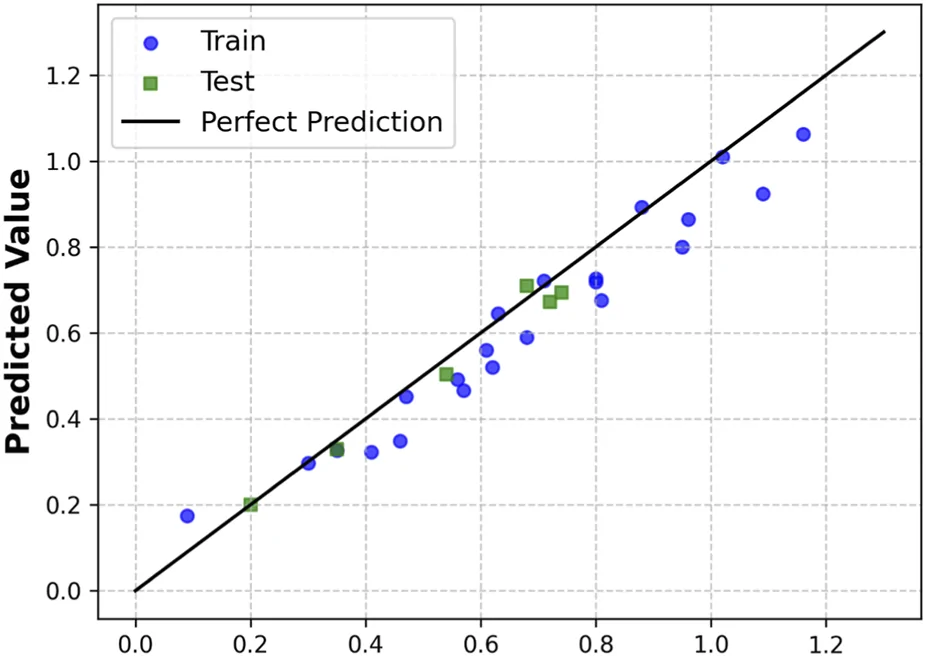
ADA-LR model: Actual Vs. Predicted values.

Adaboost combined with TSR also performs well, with a respectable R-squared score of 0.9646. While it has a higher RMSE compared to ADA-MLP, it still provides valuable predictive capabilities. Figure 7 depicts a graphical illustration of the juxtaposition between the observed values and the projected values utilizing the ADA-TSR model.

**FIGURE 7:**
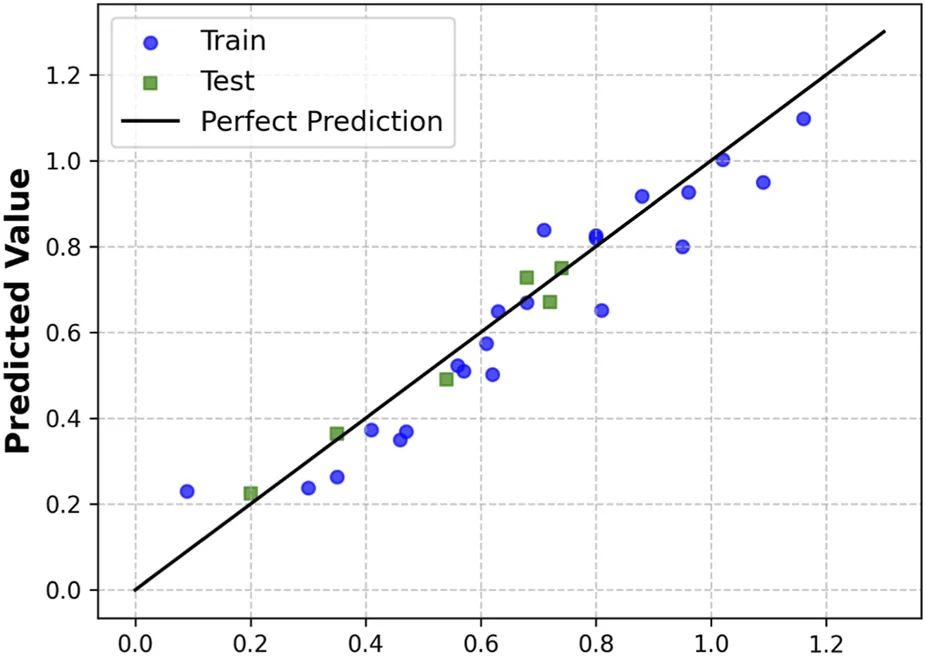
ADA-TSR model: Actual Vs. Predicted values.

The present results may also be considered in relation to conventional solubility-correlation approaches. The experimental Nystatin dataset employed here was originally reported together with empirical and semi-empirical modelling (Sajadian et al., 2023), whereas recent pharmaceutical studies have additionally applied equation-of-state and density-based thermodynamic models such as PR, SRK, and PC-SAFT (He et al., 2025; Sodeifian et al., 2025a; Zhang C. et al., 2025). In contrast, the present approach learns the temperature–pressure–solubility relationship directly from the experimental observations without requiring explicit estimation of thermodynamic model parameters. These two modelling strategies should therefore be regarded as complementary rather than directly interchangeable. Because the classical models were not re-fitted under the same train/test protocol used here, no claim of numerical superiority of the machine-learning models over thermodynamic models is made. The results suggest that the ensemble approach, Adaboost, significantly enhances model performance, and the application of GSO for hyper-parameter tuning further improves the models’ accuracy. Figures 8–11 show 3D surfaces of Nystatin ternary solubility in SCCO_2_-Ethanol for all three models. Majrashi et al. (Majrashi et al., 2024) reported similar results for Nystatin solubility. These figures are similar in general, but ADA-MLP is the smoothest. It can be clearly observed that the ADA-MLP model generates the most refined and continuous 3D surface for the solubility data of drug, which is a direct consequence of its superior predictive accuracy (*R* ^2^ = 0.99412) obtained by this model compared to other ML models. This smooth surface effectively captures the non-linear relationship between the input variables and solubility, revealing that solubility increases monotonically with both temperature and pressure within the studied range using the collected solubility dataset. The nonlinear architecture of MLP enables the model to approximate the observed interaction between temperature, pressure, and solubility without imposing a predefined functional form. The absence of abrupt fluctuations or discontinuities on this surface indicates that the smooth response surface indicates that the model provides a consistent interpolation of the observed solubility behavior within the investigated experimental domain. This smoothness is a key indicator of the model’s robustness and its ability to provide reliable predictions for process optimization. A target solubility value can then be achieved by setting pressure and temperature, while utilizing the optimized model of ADA-MLP as the best performer.

**FIGURE 8:**
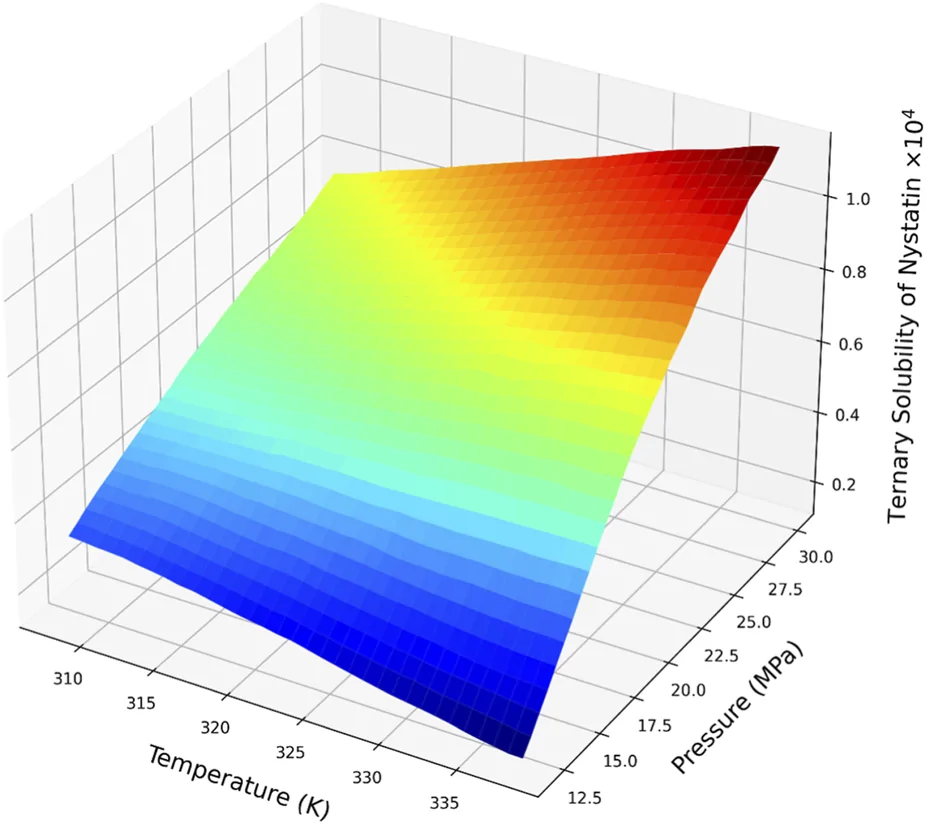
ADA-MLP model: 3D surface of ternary solubility of Nystatin in SCCO_2_-Ethanol.

**FIGURE 9:**
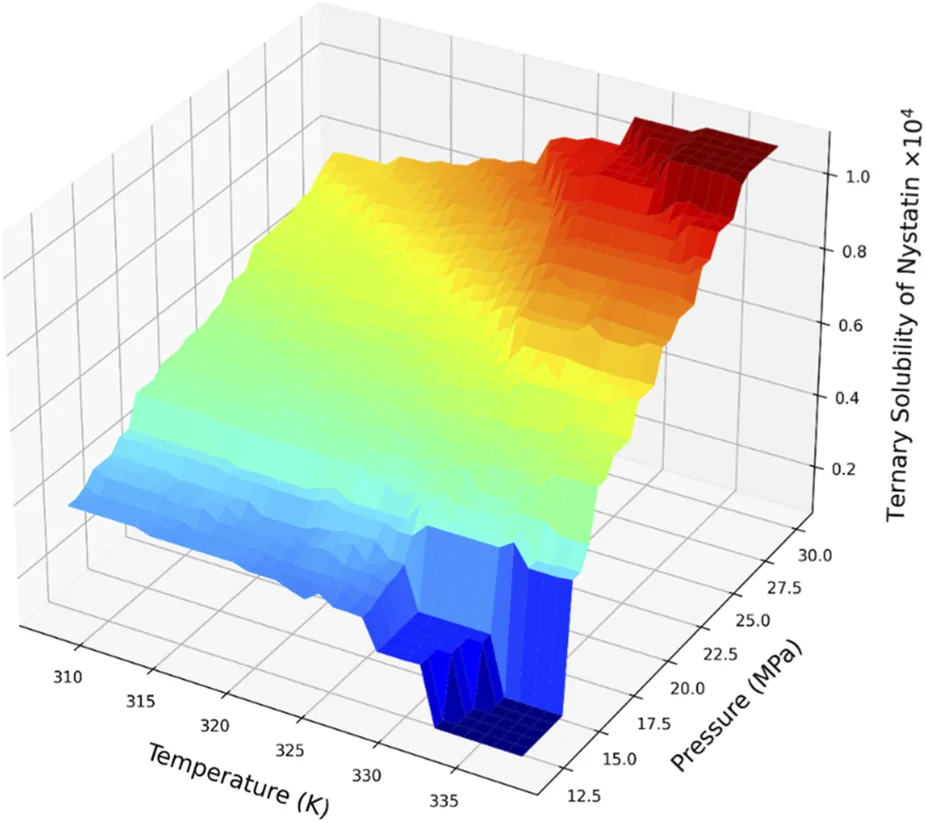
Three-dimensional response surface predicted by the ADA-KNN model for the ternary solubility of Nystatin in the SC-CO_2_–ethanol system.

**FIGURE 10:**
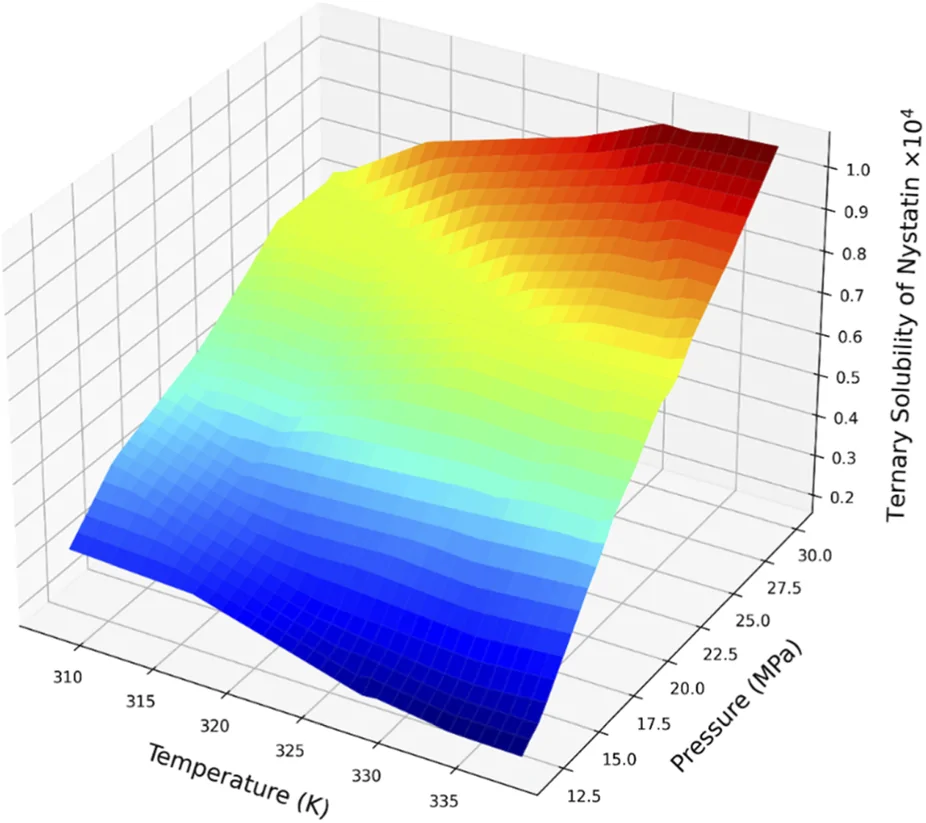
Three-dimensional response surface generated by the ADA-LR model for the ternary solubility of Nystatin in the SC-CO_2_–ethanol system.

**FIGURE 11:**
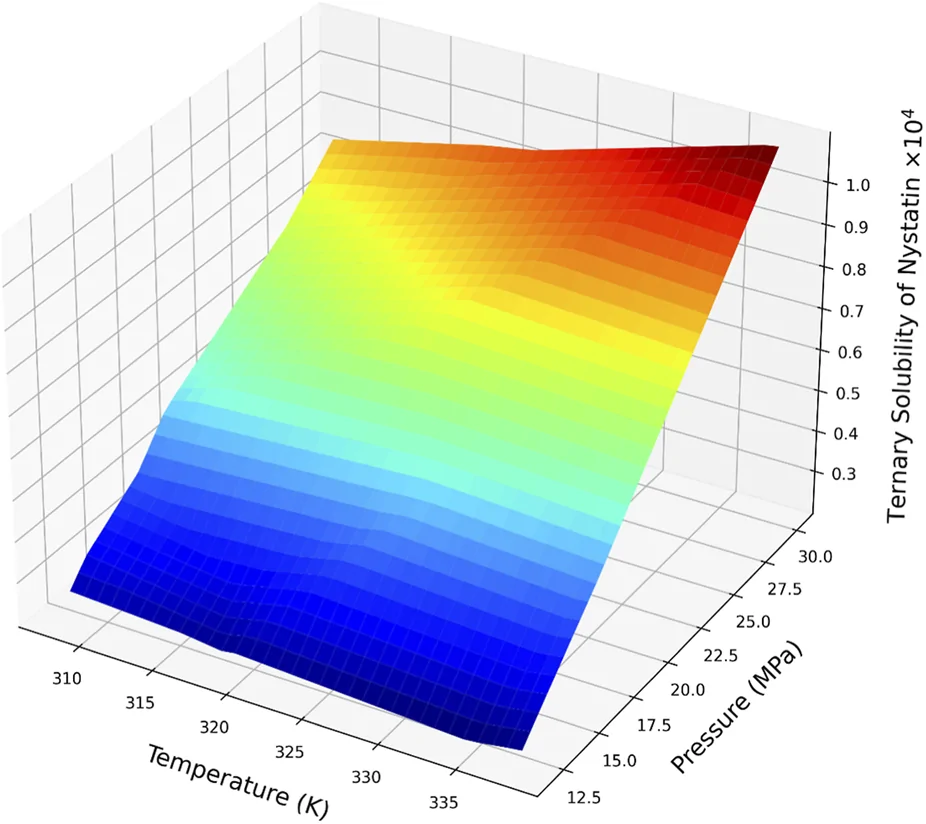
ADA-TSR model: 3D surface of ternary solubility of Nystatin in SCCO_2_-Ethanol.

In contrast to ADA-MLP, the ADA-KNN model’s 3D surface exhibits a slightly more jagged and localized structure built by the ML model, which is characteristic of its similarity-based prediction algorithm trained for the solubility dataset (Majrashi et al., 2024). The surface shows distinct “steps” or plateaus, reflecting the model’s reliance on mean the solubility near neighboring dataset. While the overall trend of increasing solubility with temperature and pressure is preserved, the surface lacks the global smoothness of the MLP model, highlighting the limitations of a local learning method in capturing a globally continuous thermodynamic function (Zhang, 2024b). This granularity suggests that while ADA-KNN is effective at interpolating within dense regions of the dataset, it may be less adept at providing smooth predictions in sparsely sampled areas. The performance metrics (*R* ^2^ = 0.97038) confirm it is a strong model, but the surface topology reveals its inherent limitations in capturing a complex, continuous physical phenomenon.

The 3D surface generated by the ADA-LR model presents a distinctly different topology, appearing as a flat plane, which is a direct consequence of its underlying linear nature. This planar surface clearly demonstrates the model’s inability to capture the curvature present in the actual data, as it forces a linear relationship between the inputs and the output. It can capture the solubility variations with pressure and temperature in the system. Despite this oversimplification, the ADA-LR model still achieves a commendable *R* ^2^ of 0.96917, indicating that the underlying solubility trend is, to a first approximation, reasonably linear. However, the surface’s failure to bend or curve suggests it has limited flexibility for representing curvature and interaction effects between the input variables. This highlights the inherent trade-off between the simplicity and interpretability of linear models and the superior flexibility of non-linear approaches for complex chemical systems. It is clearly seen that the ML model developed in this study can predict the physical behavior as well as thermodynamic phase equilibria for the ternary system with great accuracy.

The ADA-TSR model produces a 3D surface that, while following the primary trend, appears somewhat more erratic and less smooth than the surfaces generated by the MLP and LR models. This is likely due to the model’s robust, non-parametric approach, which is designed to be less sensitive to outliers by computing the median of pairwise slopes. The resulting surface, though less elegant in appearance, still provides a reliable representation of the drug solubility trend, as evidenced by its *R* ^2^ of 0.9646. The slight irregularity in the surface suggests that the model is prioritizing a more conservative fit to the overall data distribution. This makes the ADA-TSR model a valuable, albeit less aesthetically smooth, tool for process modeling.

In view of the quantitative results obtained, ADA-MLP may be regarded as the most suitable model among the evaluated alternatives for representing the ternary solubility behavior of Nystatin. On this basis, the relationships between the input variables and the predicted response are further examined through partial-dependence analysis, as presented in Figures 12, 13 (Majrashi et al., 2024; Zhang, 2024b). Zhang (Zhang, 2024b) reported similar variations in the results for Nystatin. This is a typical behavior observed for drug solubility in SC solvents as reported previously (Liu et al., 2024; Yu et al., 2026).

**FIGURE 12:**
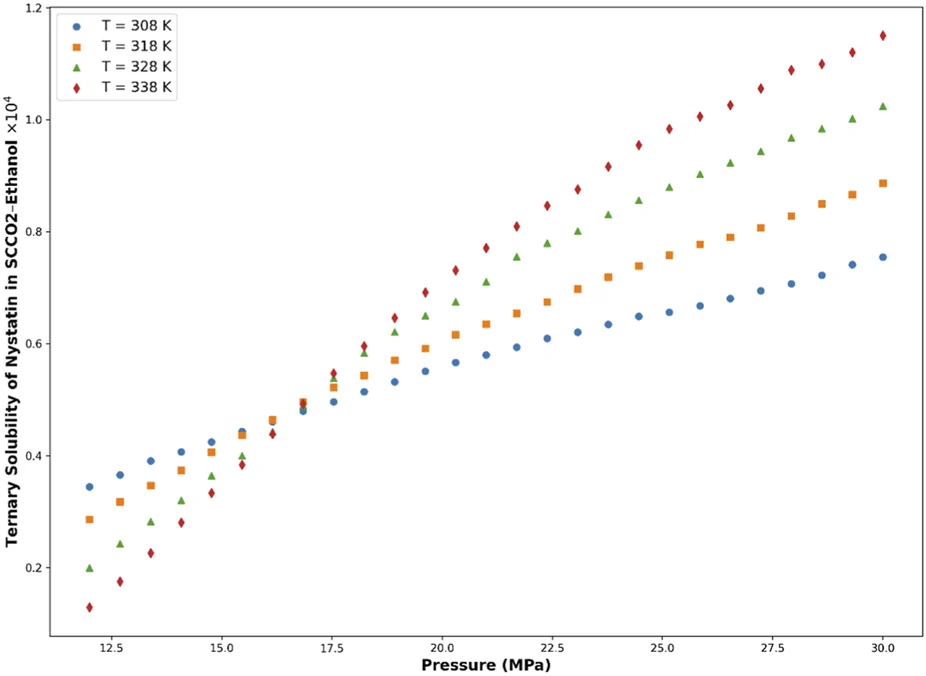
Partial-dependence plot showing the effect of pressure on the ternary solubility of Nystatin in the SC-CO_2_–ethanol system.

**FIGURE 13:**
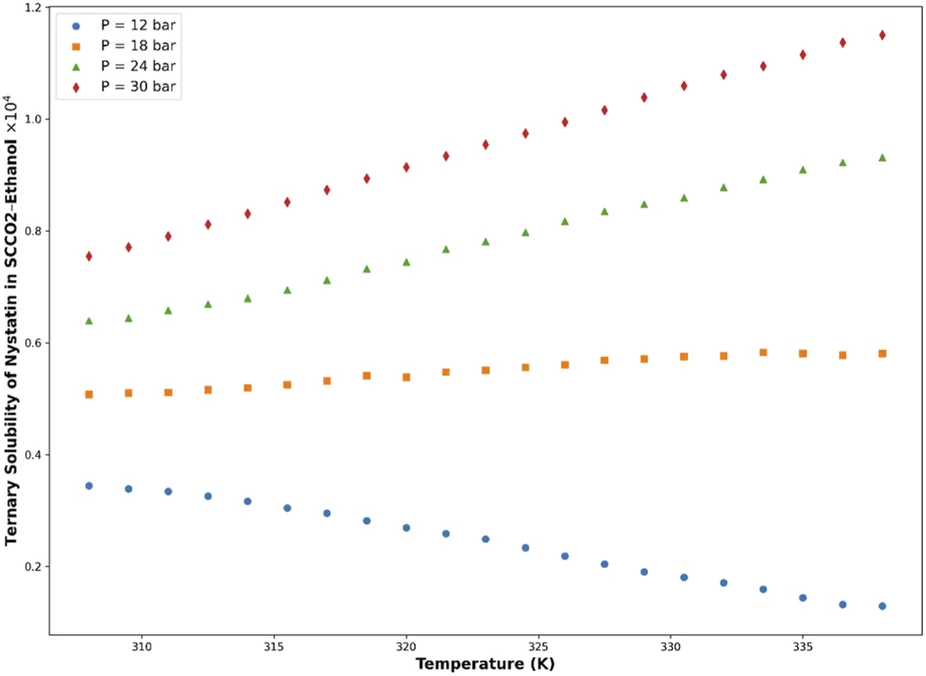
Partial-dependence plot illustrating the influence of temperature on the ternary solubility of Nystatin in the SC-CO_2_–ethanol system.

The partial dependence plot for pressure provides a clear and physically meaningful interpretation of the ADA-MLP model’s behavior, revealing a strong positive correlation between pressure and the ternary solubility of Nystatin in the binary solvent. As pressure increases, the solubility rises in an almost linear fashion, which aligns with the fundamental principles of supercritical fluid extraction where higher pressures increase the solvent density and solvating power (Meng and Liu, 2023). This trend is consistent across the entire temperature range, although the magnitude of the solubility increase is slightly more pronounced at higher temperatures. The plot’s nearly monotonic nature reinforces the idea that pressure is a primary driver for enhancing the solubility of the drug within the SCCO_2_-ethanol system. This dependency is crucial for process design, as it suggests that pressurization is an effective strategy for improving the efficiency of the supercritical fluid process.

The partial dependence plot for temperature reveals a more complex, non-linear relationship with the solubility of Nystatin, showing a distinct minimum around 315 K before increasing sharply at higher temperatures. Also, the influence of cross-over pressure point can be observed in the dataset captured by the ML model (Zhang, 2024b). This non-monotonic behavior suggests an interplay between the solvent’s density and the solute’s vapor pressure, where at lower temperatures, the density-driven solvation power is dominant, but at higher temperatures, the increased thermal energy and vapor pressure of the solute become the primary factors. The plot effectively captures the model’s ability to learn this complex, non-linear interaction from the data, a feat that would be challenging for simple empirical correlations. This characteristic minimum is a critical insight for process optimization, indicating that operating at very low temperatures could be counterproductive for maximizing solubility. Understanding this relationship is essential for selecting the optimal temperature range to achieve the desired yield in a supercritical fluid extraction or formulation process.

The pressure- and temperature-dependent trends observed in Figures 12, 13 are consistent with both the original experimental observations and the previous ML analysis of the same Nystatin–SC-CO_2_–ethanol system, which reported increasing solubility with pressure and a crossover-dependent effect of temperature (Majrashi et al., 2024; Sajadian et al., 2023). This agreement indicates that different modelling frameworks recover the same dominant response behavior; however, it should be interpreted as methodological consistency rather than independent validation because the underlying experimental source is shared.

## 5 Conclusion

Overall, the findings indicate that combining ML-based regression with GSO-driven hyperparameter optimization provides an effective computational strategy for estimating the ternary solubility of Nystatin in the SC-CO_2_–ethanol system. Our investigation reveals several key insights:

- Model Performance: The ensemble model Adaboost, in conjunction with base models, proves to be highly effective in capturing the complex relationship between Temperature, Pressure, and solubility, as evidenced by consistently high R-squared scores and low RMSE values.
- Hyper-parameter Tuning with GSO: GSO emerges as a promising approach for hyper-parameter tuning, especially in scenarios involving diverse types of hyper-parameters. Its adaptability to continuous, discrete, and categorical variables makes it a valuable tool for optimizing ML models for various domains.
- Predictive Accuracy: Among the base models, MLP (Multi-layer Perceptron) demonstrates the highest predictive accuracy, achieving R-squared value of 0.99412. This underscores the potential of neural networks in modeling complex relationships within chemical and engineering datasets.
- Real-world Applications: The accurate prediction of solubility in SCCO_2_-Ethanol based on Temperature and Pressure inputs has practical implications in chemical and pharmaceutical industries, where understanding solubility is crucial for formulation and process optimization.
- Future Directions: Further research could explore the scalability and computational efficiency of GSO when dealing with larger datasets and more complex hyper-parameter spaces. Additionally, investigating the generalizability of the ensemble model and optimization technique to other chemical systems and domains would be valuable.

In conclusion, this study demonstrates the efficacy of machine learning, GSO, and ensemble modelling techniques in effectively predicting solubility. These findings provide a strong foundation for addressing comparable challenges within the fields of chemistry and engineering. The aforementioned findings make a valuable contribution to the expanding field of predictive modelling and optimization techniques, showcasing their practical applicability across diverse industries.

## Data Availability

The original contributions presented in the study are included in the article/supplementary material, further inquiries can be directed to the corresponding author.
